# Effect of the Head Computed Tomography Choice Decision Aid in Parents of Children With Minor Head Trauma: A Cluster Randomized Trial

**DOI:** 10.1001/jamanetworkopen.2018.2430

**Published:** 2018-09-21

**Authors:** Erik P. Hess, James L. Homme, Anupam B. Kharbanda, Leah Tzimenatos, Jeffrey P. Louie, Daniel M. Cohen, Lise E. Nigrovic, Jessica J. Westphal, Nilay D. Shah, Jonathan Inselman, Michael J. Ferrara, Jeph Herrin, Victor M. Montori, Nathan Kuppermann

**Affiliations:** 1 Department of Emergency Medicine, University of Alabama at Birmingham, Birmingham; 2 Department of Emergency Medicine, Mayo Clinic College of Medicine, Rochester, Minnesota; 3 Knowledge and Evaluation Research Unit, Mayo Clinic College of Medicine, Rochester, Minnesota; 4 Division of Pediatric Emergency Medicine, Department of Emergency Medicine, Mayo Clinic, Rochester, Minnesota; 5 Division of Pediatric Emergency Medicine, Department of Pediatrics, Mayo Clinic, Rochester, Minnesota; 6 Department of Pediatric Emergency Medicine, Children’s Hospitals and Clinics of Minnesota, Minneapolis; 7 University of California Davis Health, Sacramento; 8 Division of Emergency Medicine, Department of Pediatrics, University of Minnesota, Minneapolis; 9 Division of Emergency Medicine, Nationwide Children’s Hospital, Columbus, Ohio; 10 Division of Emergency Medicine, Boston Children’s Hospital, Boston, Massachusetts; 11 Parent Representative, Rochester, Minnesota; 12 Division of Health Care Policy and Research, Department of Health Sciences Research, Mayo Clinic College of Medicine, Rochester, Minnesota; 13 Mayo Clinic Robert D. and Patricia E. Kern Center for the Science of Healthcare Delivery, Rochester, Minnesota; 14 Division of Trauma, Critical Care, and General Surgery, Department of Surgery, Mayo Clinic College of Medicine, Rochester, Minnesota; 15 Yale University School of Medicine, New Haven, Connecticut; 16 Department of Pediatrics, University of California Davis School of Medicine, Sacramento

## Abstract

**Importance:**

The Pediatric Emergency Care Applied Research Network prediction rules for minor head trauma identify children at very low, intermediate, and high risk of clinically important traumatic brain injuries (ciTBIs) and recommend no computed tomography (CT) for those at very low risk. However, the prediction rules provide little guidance in the choice of home observation or CT in children at intermediate risk for ciTBI.

**Objective:**

To compare a decision aid with usual care in parents of children at intermediate risk for ciTBI.

**Design, Settings, and Participants:**

This cluster randomized trial was conducted in 7 geographically diverse US emergency departments (EDs) from April 1, 2014, to September 30, 2016. Eligible participants were emergency clinicians, children ages 2 to 18 years with minor head trauma at intermediate risk for ciTBI, and their parents.

**Interventions:**

Clinicians were randomly assigned (1:1 ratio) to shared decision-making facilitated by the Head CT Choice decision aid or to usual care.

**Main Outcomes and Measures:**

The primary outcome, selected by parent stakeholders, was knowledge of their child’s risk for ciTBI and the available diagnostic options. Secondary outcomes included decisional conflict, parental involvement in decision-making, the ED CT rate, 7-day health care utilization, and missed ciTBI.

**Results:**

A total of 172 clinicians caring for 971 children (493 decision aid; 478 usual care) with minor head trauma at intermediate risk for ciTBI were enrolled. The patient mean (SD) age was 6.7 (7.1) years, 575 (59%) were male, and 253 (26%) were of nonwhite race. Parents in the decision aid arm compared with the usual care arm had greater knowledge (mean [SD] questions correct: 6.2 [2.0] vs 5.3 [2.0]; mean difference, 0.9; 95% CI, 0.6-1.3), had less decisional conflict (mean [SD] decisional conflict score, 14.8 [15.5] vs 19.2 [16.6]; mean difference, −4.4; 95% CI, −7.3 to −2.4), and were more involved in CT decision-making (observing patient involvement [OPTION] scores: mean [SD], 25.0 [8.5] vs 13.3 [6.5]; mean difference, 11.7; 95% CI, 9.6-13.9). Although the ED CT rate did not significantly differ (decision aid, 22% vs usual care, 24%; odds ratio, 0.81; 95% CI, 0.51-1.27), the mean number of imaging tests was lower in the decision aid arm 7 days after injury. No child had a missed ciTBI.

**Conclusions and Relevance:**

Use of a decision aid in parents of children at intermediate risk of ciTBI increased parent knowledge, decreased decisional conflict, and increased involvement in decision-making. The intervention did not significantly reduce the ED CT rate but safely decreased health care utilization 7 days after injury.

**Trial Registration:**

ClinicalTrials.gov Identifier: NCT02063087

## Key Points

**Question:**

What is the effect of a decision aid in parents of children with minor head trauma?

**Findings:**

In this cluster randomized trial of 172 clinicians caring for 971 children at intermediate risk of traumatic brain injury, the Head Computed Tomography Choice decision aid increased parental knowledge, decreased decisional conflict, and increased engagement. The intervention did not reduce the emergency department computed tomography rate but safely decreased 7-day health care utilization.

**Meaning:**

Use of a decision aid in parents of children with minor head trauma had no effect on the emergency department computed tomography rate, but improved decisional quality and safely decreased downstream health care utilization.

## Teaser

This cluster randomized trial compares the effect of the Head Computed Tomography Choice decision aid with usual care in parents of children with minor head trauma at intermediate risk for clinically important traumatic brain injuries who are treated in emergency departments.

## Introduction

Every year in the United States, over 450 000 children present to emergency departments (EDs) for evaluation of head trauma.^1^ Clinicians in the United States obtain cranial computed tomography (CT) imaging in 37% to 50% of children with minor head trauma (Glasgow Coma Scale [GCS] scores of 14-15).^2^ However, less than 10% of these CT scans show evidence of traumatic brain injury (TBI) and only 0.2% require neurosurgical intervention.^3^

To avoid unnecessary CT imaging and limit ionizing radiation exposure,^4^ the Pediatric Emergency Care Applied Research Network (PECARN) developed 2 clinical prediction rules, 1 for children younger than 2 years and 1 for children ages 2 to 18 years.^5^ Each of these prediction rules consists of 6 readily available clinical factors (eTable 1 in Supplement 1). If none of these risk factors are present, CT is not recommended. If the child has either of 2 high-risk factors, CT is recommended. If the child has 1 or 2 non–high-risk factors (those at intermediate risk), other considerations such as clinician experience, parental preference, and/or symptom progression guide the decision to obtain CT imaging. However, the PECARN rules provide little evidence to guide the choice of home observation or CT scanning in children at intermediate risk for clinically important TBI (ciTBI).

Decision aids are patient-centered tools that help clinicians and patients work together to apply the latest scientific evidence and patients’ values and preferences to care decisions.^6^ Use of decision aids has been shown to increase patients’ knowledge and involvement in decision-making and appropriately tailor testing to disease risk.^6,7^ Given the limited data available to guide CT decision-making for children at intermediate risk for ciTBI and the demonstrated effectiveness of decision aids, we designed a decision aid, Head CT Choice, and compared its effectiveness with usual care at the level of the parent-clinician dyad. Given the risk of contamination associated with randomizing at the patient level, we randomized at the clinician level.

## Methods

### Study Design

This practical cluster randomized trial^8,9^ compared an intervention group receiving a structured risk assessment and corresponding decision aid with usual care for the management of children with minor head trauma at intermediate risk for ciTBI. The study was conducted at 7 geographically diverse EDs across the United States, only 1 of which was a participant in PECARN.

### Study Population

**Figure 1.**
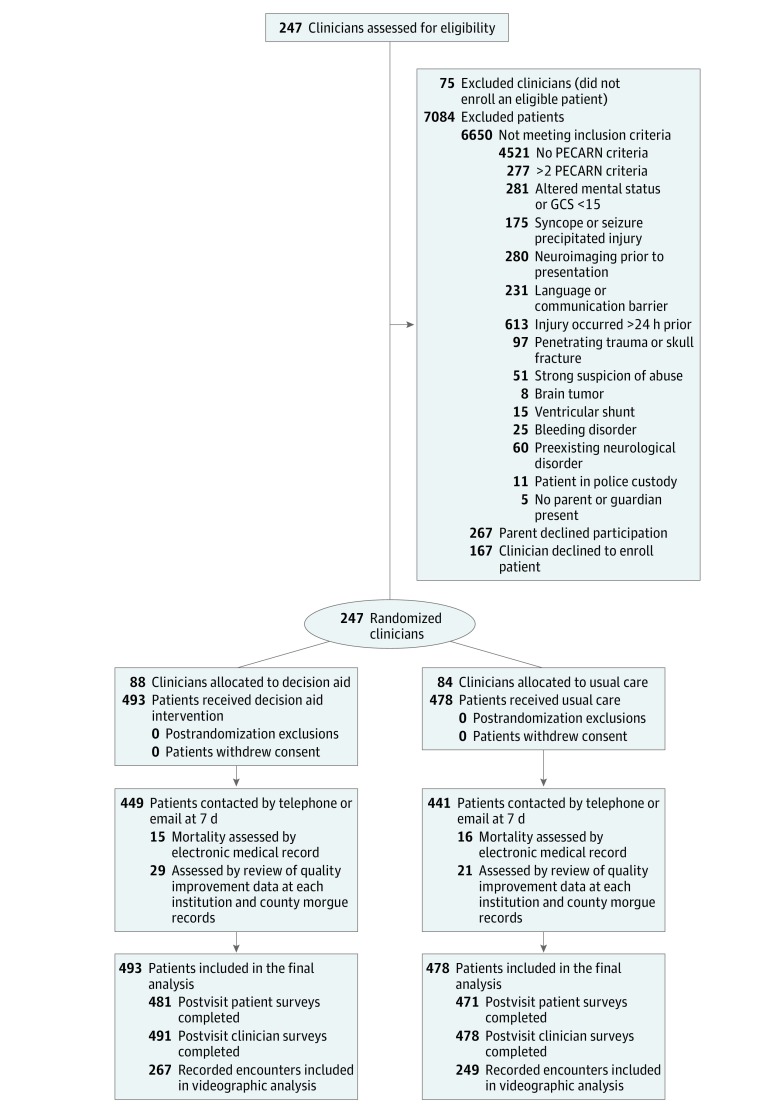
Participant Flow Diagram EMR indicates electronic medical record; GCS, Glasgow Coma Scale; PECARN, Pediatric Emergency Care Applied Research Network.

Clinicians (attending physicians, pediatric emergency medicine fellows, and advanced practitioners) caring for children with minor head trauma were eligible for cluster randomization. Eligible children were younger than 18 years and had 1 or 2 PECARN non–high-risk factors for ciTBI within 24 hours of minor head trauma, defined by a GCS score of 15 after a nonnegligible traumatic mechanism (ie, excluding ground-level falls and running into stationary objects). Children were excluded if they had a high-risk PECARN factor (GCS score <15 or other signs of altered mental status, palpable skull fracture, or signs of basilar skull fracture), 3 or more PECARN non–high-risk factors, known brain tumor, penetrating head trauma, known bleeding disorder or coagulopathy, ventricular shunt, preexisting neurological disorder complicating mental status assessment, transferred to the ED with imaging already obtained, known pregnancy, or accompanied by parents who were hearing or visually impaired, non–English speaking, or otherwise unable to use the decision aid (Figure 1).

The study protocol was approved by the institutional review board at each participating site (Trial Protocol in Supplement 2). Written informed consent was obtained from each participating clinician and parent. Assent was obtained from children ages 12 years or older. This study followed the Consolidated Standards of Reporting Trials (CONSORT) reporting guideline.

### Randomization and Masking

Given the risk of contamination associated with patient-level randomization, we randomized at the clinician level. A statistician at a centralized location performed randomization to conceal allocation. Clinicians were randomized in a 1 to 1 ratio. Randomization was stratified by site and whether their primary clinical training was in a pediatric specialty (pediatrics or pediatric emergency medicine) or another clinical specialty (general emergency medicine, family medicine, or internal medicine). We used dynamic allocation to balance randomization within strata defined by site and clinician specialty.

### Participant Identification and Enrollment

Study coordinators identified potentially eligible parent-patient dyads based on a chief complaint of head trauma recorded at the time of ED registration and through real-time communication with clinicians. Clinicians were instructed to obtain the PECARN risk factors during the initial history and physical examination but to defer discussing diagnostic options with the parent. If the clinician confirmed that the patient had 1 or 2 non–high-risk PECARN factors and met all other eligibility criteria, written informed consent was obtained to participate in the study and to video and audio record the clinical encounter.

### Study Treatments

#### Intervention

**Figure 2.**
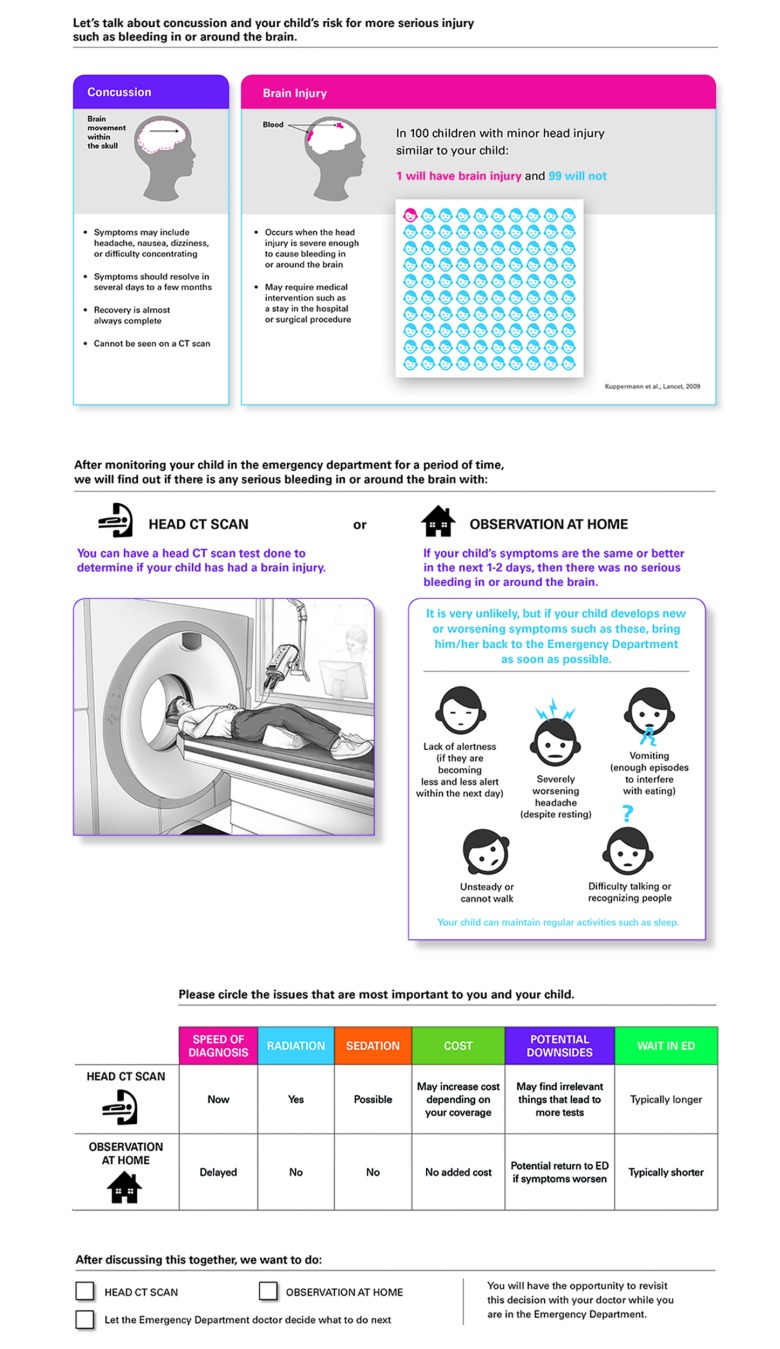
The Head CT Choice Decision Aid Decision aid used to facilitate a discussion between clinicians and parents regarding whether to obtain a cranial computed tomography (CT) scan in the emergency department (ED) or to actively observe the child at home. Data used to generate the risk of clinically important brain injury displayed on the decision aid were obtained from an investigation by Kuppermann et al.^5^ Used with permission of Mayo Foundation for Medical Education and Research. All rights reserved.

The Head CT Choice decision aid was developed in Rochester, Minnesota. Full details of this process are published elsewhere.^9^ The decision aid educates caregivers regarding the definition of a concussion and differences with other forms of TBIs, the child’s risk of ciTBI, advantages and disadvantages of cranial CT compared with active observation, and signs and symptoms that should prompt a return ED visit (Figure 2).

#### Delivery of the Intervention

Prior to the start of the trial, we calculated risk estimates for ciTBI based on the presence or absence of individual PECARN predictors in isolation, as well as combinations of predictors using the publicly available PECARN data set.^5^ The lead investigators of the study (E.P.H. and N.K.) visited each participating site and provided a 1-hour Grand Rounds presentation to participating clinicians to provide the background and rationale for the trial, without giving the details of the study intervention. Clinicians randomized to the intervention were educated separate from the Grand Rounds, and were provided information included in the decision aid and shown a video demonstrating its use. Intervention clinicians were required not to share the decision aid with other clinicians in the trial, and this was monitored by study research coordinators.

After obtaining written informed consent, a study research coordinator calculated each patient’s precise risk of ciTBI, provided the intervention clinician a decision aid corresponding with that patient’s level of risk, and offered the clinician a brief, just-in-time refresher of decision aid content and use. The clinician then brought the decision aid to the bedside and engaged parents in a shared decision-making discussion.

#### Usual Care

Study coordinators instructed clinicians randomized to usual care to discuss management options with parents according to each clinician’s usual fashion. Usual care clinicians did not have access to the precise risk estimates for ciTBI calculated from the PECARN data set or to the decision aid. As the trial was practical in design, the usual care arm was not otherwise standardized.^8^

#### Data Collection

We collected data documenting the process of screening and identifying potentially eligible participants in compliance with the Consolidated Standards of Reporting Trials (CONSORT) reporting guideline.^10^ We surveyed parents during the ED visit but before the clinical encounter to assess health literacy using the subjective literacy scale^11^ and numeracy using the subjective numeracy scale.^12^ A survey to assess parent knowledge regarding their child’s risk for ciTBI and the available management options, physician trust, decisional conflict, and the amount, clarity, and helpfulness of the information shared by the clinician was administered after the clinical encounter. We also surveyed clinicians to assess their perspective on the amount, clarity, and helpfulness of the information shared during the encounter.

We collected the following data during each enrollment or by electronic medical record review: date and time of ED registration, whether the patient was observed in the ED after the initial evaluation, specific PECARN criteria qualifying the patient for enrollment into the study, whether a CT scan was obtained, any positive findings on CT, and any return visits to the ED or findings of ciTBI within 7 days of the ED visit.

We obtained video and audio recordings of the discussions between clinicians and parents and measured the duration of the discussion from the recordings. Study coordinators contacted parents starting at 7 days after enrollment to assess health care utilization and safety. If parents were unable to be reached by telephone or email and there were no subsequent visits documented in the medical record, we reviewed process improvement reports at each participating hospital and morgue records at each participating county for any missed injuries or fatalities not identified by other methods.

### Outcomes

#### Primary Outcome

In meetings with parent representatives with prior experience in visiting EDs with their children for evaluation of minor head trauma, parent knowledge of the risk of ciTBI and the available diagnostic options emerged as the outcome of greatest importance. As a primary aim of patient-centered outcomes research is to help patients make informed health care decisions and allow their voice to be heard in assessing the value of health care options,^13^ we selected parent knowledge as the primary outcome (eFigure 1 in Supplement 1).

#### Secondary Outcomes

As a secondary outcome, we assessed the degree to which clinicians engaged parents in the decision-making process using the validated observing patient involvement (OPTION) scale.^14^ We assessed the degree of uncertainty parents experienced related to choosing between management options with which they were unfamiliar using the validated decisional conflict scale.^15^ We also measured parent trust using the validated trust in physician scale.^16^ These scales have been used with parents in prior shared decision-making studies^17,18^ (see eFigure 2 in Supplement 1 for additional information on the OPTION, decisional conflict, and trust in physician scales).

To assess health care utilization, we recorded the proportion of children who underwent CT scanning during the ED visit and any hospital, primary or specialty visits, laboratory testing, or diagnostic imaging for the 7 days subsequent to the index ED visit. Utilization data were obtained by review of itemized hospital charges on the uniform billing 92 and 04 forms (summary billing statements) and parental report at the time of the 7-day telephone follow-up. We assessed the safety of the decision aid by comparing the rate of ciTBI in each arm of the study. We used the PECARN definition of ciTBI: death from TBI, intubation for over 24 hours for TBI, neurosurgical procedure, or hospital admission of 2 nights or more for management of the head injury in association with TBI on CT.^5^

### Statistical Analysis

To estimate sample size we assumed an intraclinician correlation of ρ = 0.05 and adjusted the sample size estimates accordingly.^19^ We estimated that enrolling 950 patients would provide 99% power to detect a 16% difference in parent knowledge between the decision aid and usual care arms. This difference in knowledge was selected a priori, as it was the percentage increase in knowledge observed in a pilot trial conducted in patients in the ED setting,^20^ and there was no a priori magnitude of knowledge gain that would be considered important for the current trial. Enrolling 950 patients would also provide 95% power to determine a 15% difference in the rate of cranial CT and 82.5% power to detect a difference from a baseline ciTBI rate of 0.9% between study arms, using a 1-sided noninferiority test with an α of .05.

We analyzed all parent-child dyads in the arm to which they were randomized consistent with the principle of intention-to-treat. We compared baseline characteristics between study arms using *t* tests for continuous outcomes and χ^2^ tests for dichotomous outcomes—adjusted for clustering by clinician and stratified by study site.^19^ To test for differences in outcomes, we estimated a series of mixed-effects generalized linear models, each of which included an indicator for study group. For continuous outcomes we used linear models, and for categorical outcomes we used binomial or ordered multinomial logistic models. For the health care utilization analysis, we used the negative binomial model to measure differences in utilization. To account for nonindependence of outcomes by clinician, we included a random intercept term across clinicians in each model.

## Results

We conducted the trial from April 1, 2014, to September 30, 2016, randomizing 247 clinicians from 7 sites. There were 172 clinicians (88 decision aid; 84 usual care) who had at least 1 eligible patient. Four hundred ninety-three patients were evaluated by clinicians randomized to the decision aid and 478 by clinicians randomized to usual care (Figure 1). We recorded the parent-clinician discussion in 516 (53%) encounters. Clinician or parent refusal (n = 293) and technical difficulties with recording equipment (n = 10) were the main reasons recordings were not obtained. We contacted 890 (92%) parents by telephone or email for follow-up. Of the remaining 81 patients, 31 had follow-up visits documented in the electronic medical record in which there were no reports of complications of head injury within 7 days, and 50 had no adverse outcomes documented in the trauma registries, process improvement reports, or county morgue records at each participating site.

The mean (SD) age of the patients was 6.7 (7.1) years, 575 (59%) were male, and 253 (26%) were of nonwhite race. There were no significant differences in participant baseline characteristics between study arms (Table 1). The median (interquartile range) number of patient encounters in which intervention clinicians used the decision aid was 10 (5-16). There were 159 parents (16%) who had a high school education or less. There were no differences in parent literacy or numeracy between study arms.

**Table 1.** Comparison of Clinician and Patient Baseline Characteristics

Baseline Characteristics	No. (%)	
	Usual Care	Decision Aid
Total clinicians	84 (100)	88 (100)
Pediatric emergency medicine	52 (62)	54 (61)
General emergency medicine	21 (25)	22 (25)
Nurse practitioners/physician assistants	11 (13)	12 (14)
Total patients	478 (100)	493 (100)
Age, mean (SD), y	6.8 (5.3)	6.6 (6.7)
Age group, y		
<2	109 (23)	123 (25)
2-18	369 (77)	370 (75)
Male	285 (60)	290 (59)
Race		
White	347 (73)	371 (75)
Black	54 (11)	61 (12)
American Indian/Asian/Pacific Islander/other	77 (16)	61 (12)
Hispanic	62 (13)	54 (11)
Insurance		
Government	121 (25)	133 (27)
Commercial	278 (58)	280 (57)
Health maintenance organization	67 (14)	65 (13)
None	12 (3)	15 (3)
Annual income, $		
<20 000	66 (14)	63 (13)
20 000-29 999	26 (5)	30 (6)
30 000-39 999	34 (7)	45 (9)
40 000-59 999	55 (12)	60 (12)
60 000-79 999	51 (11)	44 (9)
80 000-99 999	60 (13)	47 (10)
≥100 000	168 (35)	185 (38)
Missing	18 (4)	19 (4)
Respondent education		
High school or less	31 (7)	27 (6)
High school or graduate education diploma	55 (12)	46 (9)
College or vocational school	126 (26)	152 (31)
College graduate, 4 y	147 (31)	151 (31)
Graduate degree	101 (21)	93 (19)
Missing	11 (2)	9 (2)
Parent present in ED		
Both parents	170 (36)	156 (31)
1 Parent	303 (63)	334 (68)
Other guardian	4 (1)	4 (1)
Literacy scale, mean (SD)^a^^,^^b^	13.6 (2.0)	13.4 (2.0)
Numeracy scale, mean (SD)^a^^,^^c^	35.7 (9.7)	36.1 (8.6)

Abbreviation: ED, emergency department.

^a^ Of the parent completing the survey.

^b^ The range of possible scores for the subjective literacy scale is from 3 to 15, with higher scores indicating higher health literacy.

^c^ The range of possible scores for the subjective numeracy scale is from 6 to 48, with higher scores indicating higher numeracy.

The PECARN risk factors for enrolled children varied by age group (eTable 2 in Supplement 1). For children younger than 2 years, severe mechanism of injury was the most common PECARN risk factor, and for those ages 2 to 18 years, any vomiting since the injury was the most common risk factor. Most enrolled patients only had 1 PECARN risk factor, with one-fifth having 2 PECARN risk factors.

### Outcomes

Parents of children cared for by clinicians randomized to the decision aid compared with the usual care arm had greater knowledge (mean [SD] questions correct out of 10: 6.2 [2.0] vs 5.3 [2.0]; mean difference, 0.9; 95% CI, 0.6-1.3) (Table 2). Parents in the decision aid arm reported less decisional conflict (mean [SD] decisional conflict score, 14.8 [15.5] vs 19.2 [16.6]; mean difference, −4.4; 95% CI, −7.3 to −2.4) and greater physician trust. Clinicians randomized to the decision aid made more effort to engage parents in the decision-making process as indicated by higher OPTION scores (mean [SD] OPTION score, 25.0 [8.5] vs 13.3 [6.5]; mean difference, 11.7; 95% CI, 9.6-13.9). Parents in the decision aid arm found the information communicated by their clinician to be of greater clarity, and they were more satisfied with the choice of whether to undergo CT scanning in the ED or to observe their child at home. A greater proportion of clinicians in the decision aid arm would recommend the way they presented information to other health care professionals (Table 2).

**Table 2.** Effect of Decision Aid on Parent Knowledge, Decisional Conflict, Trust in the Physician, Parent and Caregiver Involvement in the Decision, Patient Acceptability and Satisfaction, and Clinician Acceptability

Outcome	Decision Aid (n = 493)	Usual Care (n = 478)	Effect, Mean Difference (95% CI)	Effect, OR (95% CI)	*P* Value
Parent and/or caregiver knowledge					
Knowledge, mean (SD) No. of questions correct out of 10	6.2 (2.0)	5.3 (2.0)	0.9 (0.6 to 1.3)		<.001
Decisional conflict and trust					
Decisional conflict scale score, mean (SD)^a^	14.8 (15.5)	19.2 (16.6)	−4.4 (−7.3 to −2.4)		<.001
Trust in physician scale score, mean (SD)^b^	91.5 (11.9)	89.3 (13.7)	2.2 (0.4 to 4.1)		.02
Parent and/or caregiver involvement in the decision					
OPTION scale score, mean (SD) (n = 510)^c^	25.0 (8.5)	13.3 (6.5)	11.7 (9.6 to 13.9)		<.001
Parent or caregiver acceptability, No. (%)					
Amount of information				1.4 (0.8 to 2.5)	.29
Satisfied	455 (92)	441 (92)			
Unsatisfied	21 (4)	28 (6)			
Clarity of information				1.5 (1.1 to 2.1)	.02
Satisfied	382 (78)	342 (72)			
Unsatisfied	94 (19)	124 (26)			
Helpfulness of the information				1.4 (1.0 to 2.0)	.05
Satisfied	377 (77)	344 (72)			
Unsatisfied	101 (21)	124 (26)			
Would recommend to others				1.4 (1.0 to 1.9)	.08
Yes	376 (76)	343 (72)			
Not sure or not at all	103 (21)	126 (26)			
Would want to use for other decisions				1.4 (1.0 to 1.9)	.04
Yes	327 (66)	290 (61)			
Not sure and/or not at all	151 (31)	181 (38)			
Parent and/or caregiver satisfaction, No. (%)					
Satisfied with the choice				1.4 (1.1 to 1.9)	.02
Strongly agree	254 (52)	210 (44)			
Agree	165 (34)	199 (42)			
Not sure or not at all	46 (9)	56 (12)			
Clinician acceptability, No. (%)					
Helpfulness of the information				1.8 (1.0 to 3.3)	.05
Yes	247 (50)	192 (40)			
Not sure and/or not at all	237 (48)	278 (58)			
Present information on other diagnostic choices in the same way?				1.5 (0.7 to 3.4)	.34
Yes	281 (57)	216 (45)			
Not sure and/or not at all	207 (42)	259 (54)			
Recommend the way you presented information to other health care professionals?				2.9 (1.2 to 6.8)	.01
Yes	305 (62)	198 (41)			
Not sure and/or not at all	183 (37)	275 (58)			

Abbreviations: OPTION, observing patient involvement; OR, odds ratio;

^a^ The range of possible scores for the decisional conflict scale is from 0 to 100, where higher scores indicate increased parent uncertainty about the choice.

^b^ The range of possible scores for the trust in physician scale is from 0 to 100, where higher values indicate higher levels of trust in the physician.

^c^ The range of possible scores for the OPTION scale is from 0 to 100, where higher scores indicate greater parental engagement. The correlation coefficient between raters for OPTION scale assessments was 0.72 (95% CI, 0.67-0.76).

There was no significant difference in the proportion of patients who had CT scans while in the ED (decision aid, 22% vs usual care, 24%; odds ratio, 0.81; 95% CI, 0.51-1.27) (Table 3). Furthermore, there was no difference in the rate of CT scanning at 7 days. The ED length of stay was significantly shorter in the decision aid arm. There was no difference in the frequency of hospital admission or return ED visits within 7 days between study arms. The diagnostic discussion with parents took, on average, 2 minutes longer in the decision aid arm (mean [SD], 7.6 [0.4] vs 5.5 [0.2] minutes; *P* < .001).

**Table 3.** Effect of Decision Aid on Management and 7-Day Outcomes

Characteristic	No. (%)		Odds Ratio (95% CI)	*P* Value
	Decision Aid (n = 493)	Usual Care (n = 478)		
Cranial CT obtained in the ED	109 (22)	116 (24)	0.81 (0.51 to 1.27)	.35
Cranial CT^a^				
1 PECARN risk factor	77 (19)	73 (19)	0.96 (0.55 to 1.68)	.88
2 PECARN risk factors	32 (34)	43 (44)	0.56 (0.26 to 1.23)	.15
Cranial CT obtained within 7 d, including index ED visit	116 (24)	125 (26)	0.81 (0.50 to 1.31)	.39
ED length of stay, mean (SD), min	176 (135)	199 (162)	−22.8 (−41.6 to −4.0)^b^	.02
Admitted to the hospital	9 (2)	9 (2)	0.97 (0.38 to 2.45)	.94
ED return visit within 7 d	10 (2)	18 (4)	0.54 (0.24 to 1.24)	.15
Clinically important traumatic brain injury at 7 d	0	1 (0.2)^c^	NA	NA

Abbreviations: CT, computed tomography; ED, emergency department; NA, not applicable; PECARN, Pediatric Emergency Care Applied Research Network.

^a^ Denominator only includes patients with 1 and 2 PECARN risk factors, respectively.

^b^ Values are expressed as mean difference (95% CI).

^c^ Was diagnosed during the index hospital visit and the patient was admitted to the hospital for further management.

One patient in the usual care arm had a ciTBI. This was an infant who was acting abnormally according to the parent. The patient had an extra-axial hematoma identified on CT during the index ED visit. It was confirmed after hospital admission that the patient had sustained nonaccidental trauma. There were no missed ciTBIs in either study arm.

Seven-day health care utilization by treatment arm obtained from hospital-level billing data on all enrolled patients is shown in eTable 3 in Supplement 1. Patients of clinicians randomized to the decision aid had significantly fewer imaging procedures within 7 days of ED discharge. This difference was because of procedures other than cranial CT scans, such as cervical spine and extremity radiography. There were also significantly fewer blood tests in the decision aid arm. Unadjusted raw counts of procedures by study are shown in eTable 4 in Supplement 1.

## Discussion

In this large cluster randomized trial of children with minor head trauma at intermediate risk of ciTBI, parents who used a decision aid with their clinician had greater knowledge, less decisional conflict, greater physician trust, and greater involvement in CT decision-making. Although there was no significant difference in the frequency of CT imaging between study arms, patients of intervention clinicians underwent fewer imaging procedures and laboratory tests within 7 days. There were no missed cases of ciTBI in either study arm. To our knowledge, this is the largest multicenter trial of a shared decision-making intervention and the first to test an intervention in parents seeking emergency care for children with minor head trauma.

The magnitude of the differences in parent knowledge, decisional conflict, and parent involvement observed in this trial is similar to prior trials of encounter-level decision aids.^21,22^ These findings suggest that the decision aid improved decisional quality as intended. Parents who were engaged in CT decision-making using the decision aid had lower health care utilization at 7 days. Although the reason for this is uncertain, it is possible that parents randomized to the decision aid sought follow-up investigations less frequently after the ED visit. The PECARN prediction rules were already being used in practice at each of the participating sites before conducting the trial. Thus, the ED CT rate of 24% in the usual care arm was likely influenced by prior adoption of the PECARN rules. In our trial of shared decision-making in ED patients with chest pain in which we observed a lower rate of cardiac stress testing in the intervention arm,^7^ no prediction rule was consistently used in practice before conducting the trial. These observations suggest that encounter-level decision aids can be viewed as bundled interventions that translate evidence into practice and improve decisional quality but may variably affect health care utilization depending on the degree to which current evidence guides practice.

Patients of clinicians randomized to the decision aid also had shorter ED lengths of stay. It is possible that parents who were engaged in imaging decisions using the decision aid were in closer communication with their care team, facilitating more timely discharge. As there was no missed ciTBI in either study arm, the data suggest that the intervention is as safe as usual care. However, the trial was not powered to assess safety.

### Limitations

The main strengths of this study include a relatively large sample size, multicenter enrollment, careful measurement of decisional quality outcomes, and inclusion of robust estimates for ciTBI. The main limitations were lack of blinding, risk of contamination associated with randomizing at the clinician level, inability to obtain video recordings in all encounters, and lack of data to compare injury severity between arms. We used several approaches to mitigate these risks. Research coordinators closely monitored for contamination during each enrollment, limited decision aid access to intervention clinicians, and provided estimates for ciTBI calculated from the public access PECARN database to only intervention clinicians. Although video recordings were not obtained in all encounters, the number of videos that were obtained was sufficient to rigorously compare parent involvement in decision-making between study arms. We compared the number of PECARN risk factors in patients younger than 2 years and patient ages 2 to 18 years between arms and did not observe any significant differences. In addition, many of the participants in this trial were from higher socioeconomic status groups. As such, the results may not generalize to lower socioeconomic status populations in whom the decision aid perhaps may have greater impact.

## Conclusions

Use of the Head CT Choice decision aid in parents of children with minor head trauma at intermediate risk of ciTBIs was associated with greater parent knowledge of the risk of ciTBI and the available diagnostic options. It also was associated with less decisional conflict, greater clinician trust, and greater involvement of parents in CT decision-making. The intervention did not reduce the ED CT rate but safely decreased health care utilization at 7 days.

## Notes

Supplement 1. **eTable 1.** Clinical Factors Included in the Pediatric Emergency Care Applied Research Network (PECARN) Prediction Rules for Traumatic Brain Injury **eTable 2.** Pediatric Emergency Care Applied Research Network (PECARN) Risk Factors for the Enrolled Patients (n = 971) **eTable 3.** Healthcare Utilization Data Obtained From Hospital-Level Billing Data, Including the ED Visit and Utilization Within the Subsequent 7 Days **eTable 4.** Unadjusted Raw Counts of Procedures Obtained in Patients in Each Arm of the Trial, Including the ED Visit and Utilization Within the Subsequent 7 Days **eFigure 1.** Knowledge Questions Included in the Post-Visit Parent Questionnaire **eFigure 2.** Calculation and Clinical Significance of Differences in OPTION, Decisional Conflict, and Trust in Physician Scores **eReferences**

Supplement 2. **Trial Protocol**
