# Supplementary material for: Effect of the Head Computed Tomography Choice Decision Aid in Parents of Children With Minor Head Trauma: A Cluster Randomized Trial
Source: JAMA Netw Open. 2018 Sep 21;1(5):e182430. doi: 10.1001/jamanetworkopen.2018.2430 (PMC6324506; doi:10.1001/jamanetworkopen.2018.2430)
Supplement: Supplement 1. — eTable 1. Clinical Factors Included in the Pediatric Emergency Care Applied Research Network (PECARN) Prediction Rules for Traumatic Brain Injury eTable 2. Pediatric Emergency Care Applied Research Network (PECARN) Risk Factors for the Enrolled Patients (n = 971) eTable 3. Healthcare Utilization Data Obtained From Hospital-Level Billing Data, Including the ED Visit and Utilization Within the Subsequent 7 Days eTable 4. Unadjusted Raw Counts of Procedures Obtained in Patients in Each Arm of the Trial, Including the ED Visit and Utilization Within the Subsequent 7 Days eFigure 1. Knowledge Questions Included in the Post-Visit Parent Questionnaire eFigure 2. Calculation and Clinical Significance of Differences in OPTION, Decisional Conflict, and Trust in Physician Scores eReferences [file jamanetwopen-1-e182430-s001.pdf]

Supplementary Online Content

Hess EP, Homme JL, Kharbanda AB, et al. Effect of the head computed tomography choice decision aid in parents of children with minor head trauma: a cluster randomized trial. *JAMA Netw Open*. 2018;1(5):e182430. doi:10.1001/jamanetworkopen.2018.2430

eTable 1. Clinical Factors Included in the Pediatric Emergency Care Applied Research Network (PECARN) Prediction Rules for Traumatic Brain Injury
eTable 2. Pediatric Emergency Care Applied Research Network (PECARN) Risk Factors for the Enrolled Patients (n = 971)
eTable 3. Healthcare Utilization Data Obtained From Hospital-Level Billing Data, Including the ED Visit and Utilization Within the Subsequent 7 Days*
eTable 4. Unadjusted Raw Counts of Procedures Obtained in Patients in Each Arm of the Trial, Including the ED Visit and Utilization Within the Subsequent 7 Days
eFigure 1. Knowledge Questions Included in the Post-Visit Parent Questionnaire*
eFigure 2. Calculation and Clinical Significance of Differences in OPTION, Decisional Conflict, and Trust in Physician Scores
eReferences

This supplementary material has been provided by the authors to give readers additional information about their work.

© 2018 Hess EP et al. *JAMA Network Open*.

eTable 1. Clinical Factors Included in the Pediatric Emergency Care Applied Research Network (PECARN) Prediction Rules for Traumatic Brain Injury

**Children < 2 years of age**	**Children 2-18 years of age**
High risk factors	
GCS ≤14 or other signs of altered mental status*	GCS ≤ 14 or other signs of altered mental status*
Palpable skull fracture	Signs of basilar skull fracture
Non-high risk factors	
Occipital, parietal or temporal scalp hematoma<br>History of LOC ≥5 seconds	History of vomiting<br>History of LOC of any duration
Not acting normally per parent	Severe headache
Severe mechanism of injury†	Severe mechanism of injury†

GCS, Glasgow Coma Scale; LOC, loss of consciousness.
*Other signs of altered mental status: agitation, somnolence, repetitive questioning, or slow response to verbal communication.

†Severe mechanism of injury: motor vehicle crash with patient ejection, death of another passenger, or rollover; pedestrian or bicyclist without helmet struck by a motorized vehicle; falls of more than 0.9 m (3 feet) or more than 1.5 m [5 feet] for children 2-18 years of age); or head struck by a high-impact object.

© 2018 Hess EP et al. *JAMA Network Open*.

eTable 2. Pediatric Emergency Care Applied Research Network (PECARN) Risk Factors for the Enrolled Patients (n=971)

**PECARN risk factors***	**Usual Care (n=478)**	**Decision Aid (n=493)**
	**n (%)**	**n (%)**
**< 2 years old† (n=232)**		
Occipital, temporal or parietal scalp hematoma	23 (5)	32 (7)
Loss of consciousness >/= to 5 seconds	13 (3)	7 (1)
Severe mechanism (PECARN definition) ‡	57 (12)	83 (17)
Acting abnormally per parent	34 (7)	34 (7)
**2-18 years old† (n=739)**		
Any loss of consciousness	88 (18)	93 (19)
Any vomiting since injury	174 (37)	171 (35)
Severe mechanism (PECARN definition) ‡	98 (21)	96 (19)
Severe headache in ED	88 (18)	71 (14)
**Number of PECARN risk factors present**		
1	380 (80)	400 (81)
2	98 (20)	93 (19)

*Patients with either of the two PECARN high risk factors: GCS score ≤14 or signs of altered mental status (agitation, somnolence, repetitive questioning, or slow response to verbal communication] or signs of skull fracture were excluded.

†Participants are reported as < 2 years and 2-18 years because most children younger than 2 are preverbal, and there are two separate PECARN prediction rules for each age group, each of which has unique predictors.

‡Severe mechanism of injury: motor vehicle crash with patient ejection, death of another passenger, or rollover; pedestrian or bicyclist without helmet struck by a motorized vehicle; falls of more than 0.9 m (3 feet) (or more than 1.5 m [5 feet] for children 2-18 years of age); or head struck by a high-impact object.

© 2018 Hess EP et al. *JAMA Network Open*.

eTable 3. Healthcare Utilization Data Obtained From Hospital-Level Billing Data, Including the ED Visit and Utilization Within the Subsequent 7 Days*

Utilization Category	Decision Aid [Mean (SD)] n=493	Usual Care [Mean (SD)] n=478	Mean difference, 95% CI	p-value *
Provider Evaluation & Management codes†	1.84 (0.23)	1.88 (0.2)	-0.04 (-0.12, 0.04)	0.73
Imaging	0.65 (0.41)	0.88 (0.56)	-0.23 (-0.35, -0.11)	0.045
Blood tests	0.41 (0.32)	0.7 (0.55)	-0.29 (-0.40, -0.16)	0.046
Procedures	0.17 (0.13)	0.26 (0.19)	-0.09 (-0.17, -0.03)	0.08
Other	0.23 (0.28)	0.38 (0.46)	-0.15 (-0.26, -0.04)	0.009
Unclassified	0.02 (0.02)	0.02 (0.03)	0.00 (-0.03, 0.03)	0.80

*Adjusted data using a negative binomial model.
†Billing code assigned for services rendered by providers based on patient complexity and acuity.

© 2018 Hess EP et al. *JAMA Network Open*.

eTable 4. Unadjusted Raw Counts of Procedures Obtained in Patients in Each Arm of the Trial, Including the ED Visit and Utilization Within the Subsequent 7 Days

Utilization Category	Decision Aid [Total Number]	Usual Care [Total Number]
Provider Evaluation and Management codes*	906	897
Imaging	304	439
Tests (Total)	81	127
Procedures (Total)	211	323
Other	110	182
Unclassed	8	10

*Billing code assigned for services rendered by providers based on patient complexity and acuity.

© 2018 Hess EP et al. *JAMA Network Open*.

eFigure 1. Knowledge Questions Included in the Post-Visit Parent Questionnaire*

1. There is a possibility that my child could have bleeding in or around the brain.
2. Having a head CT scan is the only option that I have to know if my child has a brain injury.
3. A head CT is necessary to diagnose a concussion.
4. A brain injury always requires a medical intervention.
5. Having a head CT scan will confirm right away if my child has a brain injury.
6. My child will not be exposed to radiation with a head CT scan.
7. I only need to return to the Emergency Department (ED) if my child is getting worse in the next 12 hours following our discharge from the ED.
8. The CT scan may find irrelevant things that lead to more tests.
9. If my child vomits but is still able to eat, I should return to the Emergency Department.
10. I should keep my child awake for 12 hours after we leave the Emergency Department, to make sure they are ok.
11. How many children like your child do you think will have significant brain injury out of 100 children? (Provide a value of 0-100 or respond "I don't know.").

*****Questions 1-10 included the answer options "True," "False," and "Unsure." Question 11 was an open-ended question with no suggested answers.

© 2018 Hess EP et al. *JAMA Network Open*.

eFigure 2. Calculation and Clinical Significance of Differences in OPTION, Decisional Conflict, and Trust in Physician Scores

Parent engagement in the decision making process: We measured the degree to which clinicians engage parents' in decision making using the validated "observing patient involvement" or OPTION scale.[1] The OPTION scale is composed of 12 items with a value of 0-4; they are summed, divided by 48 and then multiplied by 100. This creates a score that ranges from 0-100, where higher scores are reflective of a higher level of parental engagement. Although a clinically meaningful change in OPTION score has not been defined, we anticipated that, if effective, use of the Head CT Choice decision aid would increase OPTION scores nearly twofold (compared to usual care) as observed in prior work.[2]

Decisional conflict: We measured the degree of conflict parents' experience related to feeling uninformed using the validated Decisional Conflict Scale (DCS).[3,4] The 16 items of DCS are scored on a 0-4 scale; the items are summed, divided by 16 and then multiplied by 25. The scale is from 0-100 where higher scores are reflective of parental uncertainty about the choice. A prior study found that for every unit increase in decisional conflict, patients were 19% more likely to blame their doctor for adverse outcomes.[5] For this reason, we considered a 1-unit change in the decisional conflict scale score to be clinically meaningful.

Trust in the physician: We will measure parents' trust in their clinician using the validated Trust in Physician Scale (TPS).[6] There are 9 items with a scale of 1-5, the items are subtracted by 1, summed, divided by 9 and then multiplied by 25. The scale ranges from 0-100 where higher values are reflective of higher levels of trust in their physician. To the best of our knowledge, a clinically meaningful change in trust in physician scale scores has not been published.

© 2018 Hess EP et al. *JAMA Network Open*.

eReferences

1. Elwyn G, Hutchings H, Edwards A, et al. The OPTION scale: measuring the extent that clinicians involve patients in decision-making tasks. *Health Expect.* 2005;8(1):34-42.
2. Hess EP, Hollander JE, Schaffer JT, et al. Shared decision making in patients with low risk chest pain: prospective randomized pragmatic trial. *BMJ.* 2016;355:i6165.(doi):10.1136/bmj.i6165.
3. O'Connor AM. Validation of a decisional conflict scale. *Med Decis Making.* 1995;15(1):25-30.
4. Koedoot N, Molenaar S, Oosterveld P, et al. The decisional conflict scale: further validation in two samples of Dutch oncology patients. *Patient Educ Couns.* 2001;45(3):187-193.
5. Gattellari M, Ward JE. Will men attribute fault to their GP for adverse effects arising from controversial screening tests? An Australian study using scenarios about PSA screening. *J Med Screen.* 2004;11(4):165-169.
6. Thom DH, Kravitz RL, Bell RA, Krupat E, Azari R. Patient trust in the physician: relationship to patient requests. *Fam Pract.* 2002;19(5):476-483.

© 2018 Hess EP et al. *JAMA Network Open*.
