# Supplementary material for: Effect of the Head Computed Tomography Choice Decision Aid in Parents of Children With Minor Head Trauma: A Cluster Randomized Trial
Source: JAMA Netw Open. 2018 Sep 21;1(5):e182430. doi: 10.1001/jamanetworkopen.2018.2430 (PMC6324506; doi:10.1001/jamanetworkopen.2018.2430)
Supplement: Supplement 2. — Trial Protocol [file jamanetwopen-1-e182430-s002.pdf]

Shared Decision Making in Parents of Children with Blunt Head Trauma: Head CT Choice

Investigators:
Erik Hess, MD, MSc Principal Investigator, Mayo Clinic
200 First Street SW
Rochester, Minnesota 55905
(507) 284-7221
hess.erik@mayo.edu

Nathan Kuppermann, MD MPH
University of California, Davis School of Medicine

Anupam Kharbanda, MD MS
Children’s Hospitals and Clinics of MN

Jeffrey Louie, MD
University of Minnesota Masonic Children’s Hospital

Peter Dayan, MD MSc
Columbia University College of Physicians and Surgeons

Annie Leblanc, PhD, Nilay Shah PhD, Jessica Westphal, Victor Montori MD MSc
Mayo Clinic

Daniel Cohen, MD
Tyler Arnold, MD
Nationwide Children’s Hospital Columbus, Ohio

Lise Nigrovic, MD
Boston Children’s Hospital Boston, Massachusetts

Approved: March 11, 2014

Funding Source: Patient-Centered Outcomes Research Institute (PCORI)

Contacts for Research Support

Questions Regarding:	Contact Person:
• Site enrollment<br>• Study justification<br>• Funding issues	Erik Hess, MD<br>(507) 284-7221<br>Hess.erik@mayo.edu
• Protocol<br>• IRB<br>• Patient eligibility<br>• Informed consent process<br>• Training needs	Michael Ferrara<br>(507) 293-1239<br>Ferrara.michael@mayo.edu<br><br>Derek VanMeter<br>(507) 255-4732<br>Vanmeter.derek@mayo.edu
• Statistical analysis	Jeph Herrin, PhD<br>Jeph.Herrin@yale.com
• Randomization/Registration<br>• REDCap access<br>• Reporting	Megan Branda, MS<br>(507) 266-1957<br>Branda.megan@mayo.edu<br><br>Jonathan Inselman, BA<br>(507) 538-7192<br>Inselman.jonathan@mayo.edu
• Scheduling meetings with the principal investigator	Sue Kirk<br>(507) 255-6501<br>Fax: (507) 255-6593<br>Kirk.Susan@mayo.edu

<u>Contents</u>

Section		Page
1.0	Specific Aims	3
2.0	Background and Significance	3
3.0	Preliminary Work	4
	3.1 Development of Decision Aid	4
	3.2 Preliminary study	5
4.0	Research Design and Methods	6
	4.1 Overview	6
	4.2 Schema	6
	4.3 Study setting and participants	7
	4.3.1 Emergency department participants	7
	4.3.1.1 Eligibility criteria of clinicians	7
	4.3.2 Patient selection	7
	4.3.2.1 Eligibility criteria	7
	4.4 Registration/randomization procedures	8
	4.5 Intervention	8
	4.5.1 Head CT Choice Decision Aid	9
	4.5.1.1 Training of personnel	9
	4.5.2 Usual Care	9
	4.6 Calendar of events	10
	4.7 Data collection	10
	4.7.1 Parents’ collected characteristics	11
	4.7.2 Parents’ reported outcomes	11
	4.7.3 Childrens’ clinical outcomes	13
	4.7.4 Clinician reported outcomes	14
	4.8 Statistical analysis	14
	4.8.1 Analysis plan	14
	4.8.2 Missing data	14
	4.8.3 Sample size estimations	15
	4.8.4 Allocation Process	15
	4.8.5 Healthcare utilization	16
5.0	Conflict of Interest	16
6.0	Human Subjects	16
	6.1 Study monitoring	16
	6.1.1 Data Safety Monitoring Board	16
	6.2 Early Termination	17
	6.3 Data management	17
	6.4 Video and audio recording	18
	6.5 Inclusion of Women and Minorities	18
References		19

1.0 Specific Aims

Our long-term goal is to promote evidence-based, patient-centered evaluation in the acute setting to more closely tailor testing to disease risk. As PCORI specifies, to "compare the use of risk stratification tools with usual clinical approaches to treatment selection or administration," we propose the following Aim:

- Test if the decision aid, Head CT Choice, improves validated patient-centered outcome measures and safely decreases healthcare utilization.

  Hypothesis: The intervention will significantly increase parents' knowledge, engagement, and satisfaction, decrease the rate of head CT use, and decrease 7-day total healthcare utilization with no significant increase in adverse events.

2.0 Background and Significance

Blunt head trauma is a common cause of death and disability in children worldwide and accounts for approximately 74,000 deaths and 60,000 hospital admissions in the U.S. annually.[1, 2] Each year more than 650,000 children visit U.S. EDs with apparently minor head trauma (Glasgow Coma Scale [GCS] scores of 14-15).[3]

Children with traumatic brain injuries (TBIs) who require acute neurosurgical intervention should be identified rapidly. Cranial CT is the reference standard test for the emergent diagnosis of TBI and is used to identify patients who require acute intervention.

Cranial CT for children with minor head trauma, however, is greatly over utilized. Of the 650,000 children assessed annually for blunt head trauma, approximately 35% undergo head CT. Of these, fewer than 10% have evidence of TBI on CT, and only 0.1% have an intracranial lesion requiring surgical intervention.

Over the past 10 years, CT use has more than tripled both in the U.S. and internationally. Although the benefit to the individual patient can be substantial, cranial CT exposes children to ionizing radiation which has been linked to the development of brain tumors and leukemia.[4] Of the 600,000 cranial and abdominal CT scans performed in U.S. children annually, it has been estimated that 500 might ultimately die from cancer due to CT radiation.[5] Moreover, patients may undergo multiple CT examinations over a lifetime, and the cumulative radiation dose often exceeds the 50 mSv threshold[6] that has been linked to the development of cancer among atomic bomb survivors.[7]

Although there is considerable controversy about the potential risks associated with radiation exposure from CT, all would agree that we should only use as much imaging and radiation as is necessary for patient-centered care and nothing more.

In response to the public health implications of radiation exposure from CT, the

National Cancer Institute has released a guide for health care providers that recommends immediate strategies to minimize CT radiation exposure in children.[8] Different prediction models have been developed to risk-stratify children with minor head trauma.[9-14] Earlier prediction models are limited by small sample sizes, no external validation, and/or no independent assessment of preverbal children < 2 years of age. Kuppermann, Dayan (investigators on the current proposal) and colleagues, prospectively derived and validated the Pediatric Emergency Care Applied Research Network (PECARN) clinical prediction rules for cranial CT in children with minor head trauma (one for children < 2 years and a second for children 2-18 years).[3] These prediction rules, which were developed and validated in more than 42,000 children from 25 U.S. EDs, are the most robust prediction models for minor head trauma in children and are sufficiently reliable and accurate to use in practice.[15]

No head injury prediction models, however, were specifically designed to engage the primary stakeholder – the parent/patient dyad – in the decision-making process. The conventional approach to clinical prediction rule development considers the clinician to be the primary decision maker and minimally, if at all, engages patients in decision-making. In the case of CT for minor head trauma, this is particularly troubling because parents are often unaware of their child's risk for a significant TBI, the radiation exposure associated with CT use, and the potential harmful effects of radiation when the decision is made to obtain a cranial CT or to further observe their child. We undertake the first study to intentionally educate and engage parents of children with minor head trauma in the decision making process for head CT.

3.0 Preliminary Work

3.1 Decision Aid Development

We derived and validated two clinical prediction rules to identify children at very low risk of clinically-important TBIs in more than 42,000 children from 25 U.S. EDs in the PECARN network.[3] In this study we developed 2 prediction rules – one for children < 2 years of age and another for those 2-18 years – that include specific criteria to guide clinicians in deciding whether a CT is necessary, unnecessary or indeterminate based on the presence or absence of several factors such as altered mental status, a history of loss of consciousness, the severity of the mechanism of injury, whether the child is behaving normally according to the parent, or vomiting. These rules identify high-risk children for whom cranial CTs are indicated, middle-risk children for whom either observation or cranial CT are viable options, and very low-risk children for whom cranial CTs can be safely obviated.

We also conducted a systematic review of the literature to identify tools to engage patients in shared decision making (SDM) in the ED to define the state-of-the-art and to identify knowledge gaps.[16] In our review we identified 5 decision support interventions designed to engage ED patients in SDM, three of which were for use in children. Yamamoto and colleagues presented a standardized information sheet that explained the risks and benefits of diagnostic procedures to 37 parents of children at risk for occult bacteremia.[17] In this study 92% of parents expressed a preference to be involved

in the decision-making process, and most decided to forego invasive procedures in their child (e.g., lumbar punctures). In a subsequent study that assessed 45 parents' preferences for sedation for the repair of small lacerations, all 45 parents preferred non-sedation over sedation, and 98% expressed a preference for involvement in decision-making for their child.[18] Finally, Karpas and colleagues assessed parental preferences for rehydration method in children with vomiting and diarrhea.[19] Of the 266 parents who participated in the study, most (62%) preferred intravenous to oral rehydration. *None of these decision support interventions were designed for use in parents of children with minor head trauma.* Overall, these 3 studies indicate that the overwhelming majority of parents prefer to be involved in medical decision-making for their child and that management decisions are sensitive to informed parental preferences.

Based on the above information and with stakeholder (clinicians, parents and experts in shared decision making) involvement, a decision aid for use in parents of children with minor head trauma was developed. The decision aid, *Head CT Choice* (**Appendix 1**), educates parents regarding the definitions of and difference between a concussion and a TBI, how the clinician determined the severity of their child's head trauma, their child's quantitative risk for a clinically-important TBI, the pros and cons of cranial CT compared to active observation, and what signs and symptoms parents should watch for in the next 24 hours that should prompt a return visit to the ED.

3.2 Preliminary Studies

Our preliminary work includes the conduct of a 204-patient single site randomized trial of the *Chest Pain Choice* decision aid in adults presenting to the ED with a primary complaint of non-traumatic chest pain.[20] These patients had no ischemic changes on the initial ECG, negative initial cardiac troponin testing, no history of coronary artery disease, and were being considered for admission and cardiac stress testing within 24 hours (0-6% quantitative pretest probability of ACS within 45 days). Data collection included video-recording of the patient-clinician encounter (degree of patient engagement assessed using the validated OPTION scale), chart review, post-visit patient and clinician surveys, and structured 30-day phone follow-up to assess for a major adverse cardiac event. A patient was considered to have a major adverse cardiac event if they had an acute myocardial infarction, a sustained ventricular dysrhythmia, cardiogenic shock, or death attributed to a cardiac or unknown cause within 30 days of discharge. Compared to usual care, patients in the SDM group had greater knowledge, experienced less decisional conflict related to feeling uninformed as indicated by lower decision conflict scores, and were significantly more engaged in the decision-making process as indicated by higher scores on the validated Option scale (see Table 1). Compared to patients in the control group, patients randomized to the decision aid had a 19% lower rate of admission for cardiac stress testing (58% vs. 77%, $p < 0.001$), a 16% lower rate of cardiac stress testing at 30-days (75% vs. 91%, $p = 0.002$), and there were no major adverse cardiac events after discharge in either group. These preliminary data suggest that SDM is feasible in patients at risk for an emergent diagnosis and that clinicians can effectively engage patients in SDM even in a time-limited acute care environment like the emergency department. For these reasons, we believe engaging

parents of children with minor head trauma in the decision to obtain a head CT is feasible and likely to improve both patient-centered outcomes and healthcare utilization.

Table 1: Results from *Chest Pain Choice* single center trial.

**Characteristic**	**Decision Aid (n = 101) N (%)**	**Usual Care (n = 103) N (%)**	**P-value or Mean Difference (95% CI)**
**Patient knowledge**			
6 Knowledge Questions	3.6 (3.4, 3.9)	3.0 (2.7, 3.2)	0.67 (0.34, 1.0)
Correctly assessed 45-day risk for ACS	24 (25%)	1 (1%)	<.0001
**Decisional Conflict and Trust**			
Decisional conflict scale	22.3 (18.1, 26.4)	35.9 (32.2, 39.6)	-13.6 (-19.1, -8.1)
Trust in physician	83.4 (79.4, 87.3)	79.3 (75.4, 83.2)	4.1 (-1.4, 9.6)
**Patient participation**			
Patient engagement by the clinician (OPTION scale)	51.4 (49.7, 53.0)	32.0 (31.0, 33.1)	19.3 (17.4, 21.2)

4.0 Research Design & Methods

4.1 Overview

We will conduct a multicenter cluster randomized control trial comparing the efficacy, safety and patient-centered outcomes of the shared decision-making decision aid 'Head CT Choice' to usual care among children at low-moderate risk of clinically-important TBI in the ED for whom a CT is being considered to engage parents in shared decision-making.

4.2 Overall Schema

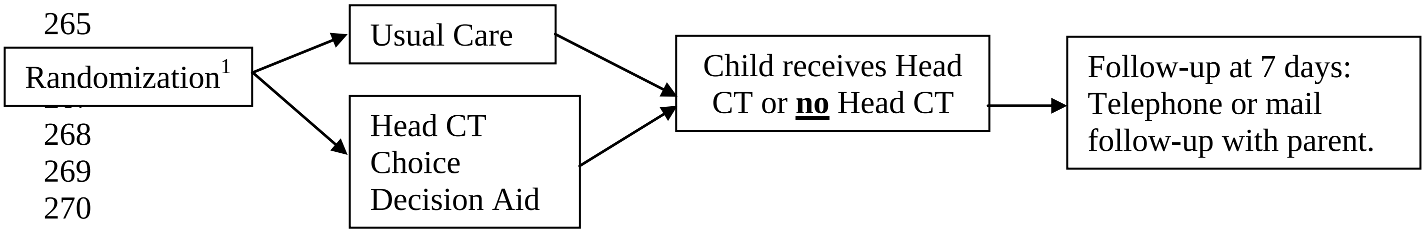

1 – Clinicians will be randomized to usual care or the Head CT Choice decision aid prior to treating an eligible patient. A detailed description of the process by which eligible patients will be identified for inclusion in the study is included in Appendix 2.

4.3 Study Setting and Participation

4.3.1 Emergency Department Setting

The locations include 4diverse hospital EDs across Minnesota – (1) Mayo Clinic in Rochester, MN (academic ED that serves a largely rural population); (2) University of Minnesota Masonic Children's Hospital in Minneapolis, MN (academic ED that serves a largely urban population); (3) Children's Hospital and Clinics of Minnesota, Minneapolis ED; and (4) Children's Hospital and Clinics of Minnesota, St. Paul ED (2 pediatric community EDs that serve largely urban populations) and 3 hospitals outside the state, (1)University of California Davis Children's Hospital, Sacramento, CA (academic ED that serves a largely urban population) (2) Children's Nationwide Hospital in Columbus, Ohio (3) Boston Children's Hospital in Boston, Massachusetts.

4.3.1.1 Eligibility Criteria of Clinicians

Clinicians caring for children with head trauma.

4.3.2 Patient Selection

Each criterion must be addressed and documented in the patient's case report form for eligibility assessment prior to randomization. No waivers or exemptions to any eligibility criteria are permitted.

4.3.2.1 Eligibility criteria

Inclusion:

Parents seeking care for a child who is:

1. < 18 years of age.
2. ≤ 24 hours since injury resulting in head trauma.
3. Moderate risk (0.9%) for clinically-important TBI according to the PECARN prediction rules.

We will exclude parents of children with:

1. GCS scores < 15
2. Evidence of penetrating trauma, signs of basilar skull fracture, or depressed skull fracture on physical examination
3. Brain tumors
4. Ventricular shunts
5. Bleeding disorder
6. Pre-existing neurological disorders complicating assessment
7. Neuroimaging at an outside hospital before transfer
8. Signs of altered mental status (agitation, somnolence, repetitive questioning, or slow response to verbal communication)
9. Syncope or seizure disorder preceded (led to) head trauma or seizure post head trauma
10. Known to be pregnant
11. Communication barriers such as visual or hearing impairment that may preclude use of the decision aid.

12. Strong suspicion of abuse for this head injury

4.4 Registration/Randomization Procedures

<u>The flow and process of identifying eligible patients for enrollment in the study is depicted in **Appendix 2.**</u>

Given the relatively limited number of pediatric clinicians at each site and the great degree of familiarity with the PECARN head CT decision rules across the specialty of pediatric emergency medicine, randomizing at the patient level is associated with a significant risk of contamination of the intervention. As such, we will randomize at the clinician level to limit contamination.

Informed consent will be obtained from clinicians at each site prior to enrollment of that clinician's parent/child dyad. After clinician consent is completed, the site study coordinator will contact the statistical team (Branda/Inselman) with enrollment information via email. The statistician will centrally randomize the clinician and communicate that to the site principle investigator and study coordinator.

Informed consent will also be obtained from the parent/guardian of a child with minor head trauma. The parent must provide signed and dated consent for the use of their Protected Health Information (this may be incorporated into the informed consent document). Prior to registering a parent(s) to the study, all of the eligibility criteria on the eligibility checklist must have been met. **No waivers or exemptions to any eligibility criteria are permitted.** All eligibility criteria must be fully documented on the case report form and subsequently entered into the study database.

4.5 Intervention

Parents/Guardians in the intervention group will discuss testing to diagnose their child's head injury with their clinician, facilitated by use of the Head CT Choice decision aid.

4.5.1 Head CT Choice Decision Aid (**Appendix 1**)

For patients whose clinician is randomized to the decision aid arm:

1. The study coordinator, using the PECARN risk estimates, will select the correct pre-printed and individualized decision aid for the parent/clinician dyad.
2. The study coordinator will provide a color-printed copy of the decision aid to the clinician prior to the clinician having the head CT discussion with the parents.
3. The study coordinator will offer to provide the treating clinician a concise refresher of the content included in the decision aid in the context of the trial.
4. The clinician will then, using the decision aid as a tool to facilitate discussion, educate the parents regarding the difference between a

concussion and a traumatic brain injury, how the clinician determined the severity of their child’s head trauma, their child’s quantitative risk for a clinically significant brain injury, the pros and cons of head CT compared to active observation, and what signs and symptoms parents should watch for in the next 24 hours that should prompt a return visit to the ED.

5. The clinician will then engage the parents in a shared decision regarding the option of head CT versus active observation and come to a decision that is consistent with both the parent’s values and preferences and the clinician’s level of comfort.

4.5.1.1 Training of Personnel

Study personnel from the central site will conduct a 1 hour grand rounds presentation and do a demonstration in the use of the decision aid during in-person visits with participating sites. Study personnel may also provide a reminder of how to use the decision aid as needed or in response to deviations in the quality of delivery observed on video recordings. Brief video clips that demonstrate the basic use of decision aid will be provided to clinicians to review at their convenience. Designated site staff will receive training in the consenting procedures prior to actually consenting patients.

4.5.2 Usual Care

For parents whose clinician is randomized to the usual care arm, the clinician will discuss management options with the parent in the clinician’s usual fashion. The clinician-parent discussion will be video-recorded, and immediate post-visit surveys to the patient and clinician administered. Patients in the usual care arm will also be contacted at 7 days to assess the study outcome measures.

4.6 Calendar of Events

Parent Forms/Schedule	Prior to Study Enrollment	Prior to discussion[1]	Discussion[2]	After discussion[1]	7 Day Follow-Up
Approached[3]	X				
Informed Consent	X				
Pre-Encounter Survey[4]		X			
Post-Encounter Survey				X	
Phone Survey					X
EMR Review					X

1- Discussion is the encounter between the clinician and the parent where the child's risk and parents' options for screening are discussed.
2- When consent is provided by both the parent and clinician the encounters will be video and/ or audio recorded.
3- Parents enrolled will be captured in the remote data capture system. Parents found to be ineligible or who declined participation will be assigned a generic study ID by the study coordinator and the reason for ineligibility or that the parent(s) declined will be captured in a tracking log.
4- The pre-encounter survey may be administered after the discussion, if the flow of care for the enrolled parents' child does not allow for it to occur prior to the discussion of interest. These events will be captured in the remote data capture system.

Clinicians Forms/schedule	Prior to Enrollment of Eligible Parents	Randomization	After discussion with each enrolled parent
Informed Consent	X		
Clinician Survey			X

4.7 Data collection

To assess the reach of the trial, study coordinators will record parents who are enrolled as well as those who were assessed for inclusion. The criteria for inclusion that the parents' child did not meet will be captured along with declines by the parent. This will allow us to measure participation and representativeness of the trial.

The enrolled parents' data will be collected via surveys prior to and after the encounter with the clinician where the disposition discussion took place. A study coordinator will collect data from the EMR for registration/randomization, and all subsequent data from time of initial visit for head trauma to 7 days after for follow-up to assess utilization and safety of the child of the enrolled parent(s). The types of resource utilization captured during the 7 day follow-up include: imaging data, outpatient visits and procedures, and inpatient hospitalizations (including admission and discharge dates, reason for admission, neurosurgical procedures, and discharge destination). The study coordinator will also obtain the PECARN risk estimate for each child whose parents provide consent. Parents will be provided a form on which to document this information at discharge from the ED or the hospital. This will allow for a more standardized collection of these data. We will also request that parents save all their healthcare bills that they receive during this 7-day period. The parent will be contacted 7 days after enrollment via phone (primary method of contact) for assessment of utilization and safety events. If the primary method of contact is not successful, the study coordinator will subsequently contact the parent(s) utilizing a secondary method

of contact that was obtained at time of consent (email, a secondary phone number, or mail) for completion of the 7-day assessment. Clinician's will be surveyed after each encounter with an enrolled parent.

Video/audio and audio recordings of the encounter will be collected, and the recordings will contain specifically the discussion of interest between the parents and the clinician regarding the results of the diagnostic investigations and management options along with the decision and actions that will carried out. Criteria for an encounter to be recorded include consent from the parent, assent from the child, and consent from the clinician for recording. If the parent(s) or the clinician decline to be video recorded, we will request that an audio recording be obtained. Either the parent(s) or clinician can decline the audio recording or stop the recording at any time during the encounter. Regardless of whether video or audio recording are obtained, all other data on enrolled parents and their child and participating clinicians will be collected, and both the clinician and parent will be retained in the trial consistent with their consent to participate and intention to treat principles.

4.7.1 Parents' collected characteristics

Preference: Parents will identify their preference in decision making style for their child prior to the encounter of interest with their treating clinician (Control preference scale).[21] There are 5 levels of preference that parents choose from and that will be reported as a count and frequency for each item.

Health literacy: Parents will respond to 3 items that assess their health literacy prior to the encounter of interest with the treating clinician. The item 'How confident are you filling out medical forms by yourself?' will be classified into inadequate (response: not at all, little bit or somewhat) versus adequate health literacy (response: quite a bit or extremely). The other two items will be reported as frequencies of raw responses without categorization.

Subjective numeracy: Parents will respond to an 8-item questionnaire prior to the encounter of interest to assess their subjective numeracy (Subjective Numeracy Scale). All 8 questions will be summed (reversing the scale for the question on percentages and words) and averaged creating an overall score ranging from 1-6 where higher scores are indicative of higher levels of numeracy.

Demographics: Parents will self-report their ethnicity/race, annual income and highest education level obtained at time of enrollment.

4.7.2 Parents reported outcomes

Decision: Parents' will report the decision made regarding their child's screening options.

Knowledge: In discussion with our parent representative and ED Patient Advisory Council, it became clear that a major focus of many parents of children with head injury will be whether their child experienced a concussion. It became apparent that the issue of whether the child has experienced a concussion is a lower priority to clinicians than whether the child has suffered a more serious structural brain injury, which is what can be detected by cranial CT. To meet the informational needs of parents, the decision aid defines and distinguishes a concussion as symptoms experienced by the child (such as headache, concentration, memory, judgment, balance and coordination) observed by others that can be diagnosed based on the history and physical examination alone, and a significant brain injury as structural damage to the brain resulting in blood collecting in spaces of the brain. Based on this design feature of the decision aid, we will assess parents' knowledge regarding the difference between a concussion and a traumatic brain injury. We will also assess parents' knowledge regarding their child's quantitative risk for a significant brain injury, the pros and cons of head CT compared to active observation, and what signs and symptoms parents should watch for in the next 24 hours that should prompt a return visit to the ED. Knowledge will be measured by means of a post-visit survey delivered immediately after the clinical encounter. Each knowledge question will provide the parent(s) with three options to respond (True, False, or Unsure), and the parent(s) will receive a score of 1 for a correct response and 0 for incorrect where any response of 'Unsure' will be considered incorrect. An overall score will be calculated by summing the correct responses and dividing by the number of questions asked. Surveys where parents' choose to not respond to any survey questions will be considered as missing. If at least one knowledge question is answered then the parent(s) will be considered as a responder and assessed for this outcome, where non-responses will be coded as incorrect.

Parent engagement in the decision making process: We will measure the degree to which clinicians engage parents' in decision making using the validated OPTION scale.[22] The OPTION scale will be assessed by having 2 observers at the central coordinating site independently review and score 20% of the videos and upon agreement being found they will divide up the remaining videos of the encounters and review independently. The OPTION scale is composed of 12 items with a value of 0-4; they are summed, divided by 48 and then multiplied by 100. This creates a score that ranges from 0-100, where higher scores are reflective of a higher level of parental engagement.

Fidelity: We will measure the degree to which the intervention is implemented as intended in both intervention and control groups when reviewing the recordings. The recordings in the intervention group will serve as a measure of the fidelity with which the intervention was delivered as intended. We will use a checklist of elements present and absent for quantification of implementation.

Decisional conflict: We will measure the degree of conflict parents' experience related to feeling uninformed using the validated Decisional Conflict Scale (DCS).[23, 24] The 16 items of DCS are scored on a 0-4 scale; the items are summed, divided by 16 and then

multiplied by 25. The scale is from 0-100 where higher scores are reflective of parental uncertainty about the choice. There are 5 DCS subscales, where a DCS subscale consists of 3 questions (1 subscale of 4). If 2 of 3 (or 3 of the 4) questions within a subscale have responses then the parent would be considered as a responder and a score could be calculated. If more than one response per subscale is missing then that specific subscale is not calculated for the parents'. An overall DCS score can be calculated if no more than 5 responses are missing as long as each missing response falls into a different subscale.

Trust in the physician: We will measure parents' trust in their clinician using the validated Trust in Physician Scale (TPS).[25, 26] There are 9 items with a scale of 1-5, the items are subtracted by 1, summed, divided by 9 and then multiplied by 25. The scale ranges from 0-100 where higher values are reflective of higher levels of trust in their physician.

Satisfaction: We will assess parents' satisfaction by asking 5 questions regarding the acceptability and satisfaction regarding the way information was shared during the encounter using a 7-point Likert .

4.7.3 Childrens' clinical outcomes

Proportion of children who undergo cranial CT: The study coordinator enrolling the parent(s) will ascertain this in real time and confirm the data by health record review of the child of the enrolled parent(s).

Healthcare utilization: We will assess healthcare utilization for the subsequent 7-days after the ED visit. Healthcare utilization will include measures such as hospitalization, re-hospitalization, primary and specialty visits, and diagnostics including CT use which will be obtained via a health record review, review of itemized hospital charges on the UB-92 and UB-04 forms (summary billing statements), and parental report via the 7 day follow-up by the study coordinator. The parent self-report will capture any ED readmissions, outpatient and subspecialty visits, the number of each type of outpatient visit, imaging data, procedures, and inpatient hospitalizations (including admission and discharge dates, reason for admission, neurosurgical procedures, and discharge destination). To assist parents in collecting utilization data, we will provide a form to document this information at discharge from the ED or the hospital. This will allow for a more standardized collection of these data if parents receive care at a location other than the primary institution. We will also identify patients who receive a significant portion of their health care at the participating centers. For these patients, we will compare the patient reported utilization to the data from each institution's electronic medical record and administrative billing data, where available and feasible.

Safety: We will assess safety by comparing the rate of clinically-important TBI in each arm of the study. We will define clinically-important TBI as we did in our prior study:[3] death from TBI, intubation for more than 24 hours for TBI, neurosurgical procedure, or hospital admission of 2 nights or more associated with TBI on CT. We defined this outcome to exclude brief intubations for imaging or overnight admission for minor CT

findings. We sought a meaningful measure for clinical decision-making which also accounted for the imperfect specificity of CT (i.e., false positive scans that might result in overnight admissions). Site investigators, blinded to ED data, will verify outcomes by medical record review. CT scans will be obtained at the ED clinician's discretion with helical CT scanners and interpreted by site faculty radiologists. A faculty radiologist, unaware of clinical data, will make definitive interpretations of inconclusive CT scans. Study coordinators will contact parents of children with minor head trauma starting at 7 days to ensure that no outcomes are missed. Medical records and imaging results will be obtained if a missed TBI is suggested at follow-up. If a ciTBI is identified, the patient's outcome will be classified accordingly. If we are unable to contact the patient's guardian by telephone or any secondary means of contact provided at the time of consent, we will review the medical record, emergency department/trauma process improvement records, and county morgue records, to ensure that no discharged patient is subsequently diagnosed with a ciTBI.

4.7.4 Clinician reported outcomes

Satisfaction: We will assess clinicians' satisfaction by asking questions regarding the helpfulness and satisfaction with the way information was shared during the encounter using a 7-point Likert scale.

Preference: Clinicians will identify their preference in decision-making style after the encounter with each enrolled parent(s) (Control preference scale).[21] This scale contains 5 levels which will be reported as frequencies and counts along with correlation to the patients reported control preference scale.

4.8 Statistical Analysis

4.8.1 Analysis Plan

We will conduct the study according to the intention to treat principle, including all parent-children dyads in the arm to which they were randomized, regardless of whether they received the intervention assigned. We will adhere to the CONSORT guidelines to transparently report study results and ensure that sufficient information is included to allow for assessment of the study's internal and external validity.

We will compare outcomes between study arms using t-tests for continuous outcomes and χ^2 tests for dichotomous outcomes, adjusted for clustering by clinician and stratified by study site[27]. If there are differences in baseline characteristics between the two study groups, these will be accounted for using hierarchical generalized logistic or linear regression models that include an indicator for study arm[28].

We will perform descriptive analyses to describe any potential heterogeneity of treatment effect (HTE) and facilitate synthesis of subgroup results in future meta-analyses. We will conduct descriptive HTE analyses by age, gender, parent race/ethnicity, GCS score, and level of parental education (collected in post-visit surveys). The outcomes assessed with HTE analyses will be the same as those assessed in the trial (e.g., patient-centered outcomes such as parental knowledge, engagement,

satisfaction and healthcare utilization). We will also conduct interaction testing to determine the interaction between the decision aid and each pre-specified patient characteristic.

4.8.2 Missing data

We will make every effort to minimize missing data. Trial enrollment and the fidelity of follow-up procedures will be reviewed during monthly conference calls. In our pilot trial of the Chest Pain Choice decision aid only 2% of patients were unable to be contacted by phone at 30 days for follow-up, indicating that study data were 98% complete. A study biostatistician will conduct frequency reports to assess for missing data, and the study team, which is experienced in conducting multicenter trials, will trouble shoot any problems encountered. Patients with missing outcome data will not be included in the assessment of that outcome. We will report rates of missing data for each outcome by study arm and known reasons for missing data. For data elements that are used to adjust study comparisons we will use multiple imputation to account for any that are missing at random (MAR).

4.8.3 Sample Size Estimation

Patient knowledge will be the primary outcome, as it was considered to be the most important endpoint by our patient representatives on the ED Patient Advisory Council. In order for the study findings to maximally impact practice and policy, however, we will need to be adequately powered to detect differences in the outcomes of interest to each of the stakeholders.

Accounting for a lost to follow-up rate of as high as 5% (there was 2% lost to follow-up in our pilot trial), approximately 75 clinicians (assuming an average of 15 clinicians/site) will enroll 950 patients. This will provide the following power to detect differences in each of the patient and stakeholder-important outcomes (using a 2-sided hypothesis test and an alpha of 0.05):

**Outcome (n = 950)**	Usual Care*	Decision Aid*	Difference	Power
**Parent knowledge**	44%	60%	16%	>99%
**Parent engagement in the decision making process**	7.0 (5.5)	27.0 (8.2)	20.0	>99%
**Decisional conflict** †	36 (19)	21 (21)	15	>99%
**Trust in the physician**	79 (20.0)	84 (20.0)	4.1	86%
**Parent satisfaction with the decision made (% agree or strongly agree they are satisfied)**	70.0 (26)	80 (26.0)	10.0	>99%
**Safety (clinically-important traumatic brain injury)** ‡	0.9%	0.9%	0%	82.5%
**Proportion of children who undergo head CT** §	49%	34%	15%	95%
**Healthcare utilization**	8.3 (0.8)	6.8 (0.7)	1.5	>99%

*Estimates were determined from our completed randomized trial.[20]

†Lower decision conflict scores indicate less conflict experienced by patients related to feeling uninformed.
‡ Noninferiority, 1-sided test with alpha = 0.05 with a maximum difference of 2%. Baseline rates determined from the PECARN prediction rule study.
§ Obtained from the rate of CT in the moderate risk group of patients in the PECARN prediction rule study.

4.8.4 Allocation process

Clinicians will be randomized at the beginning of the study and, if necessary, when first treating an eligible patient. Clinician randomization will be stratified by study site and according to whether they are pediatric emergency clinicians or not. To maintain balance the randomization algorithm utilized will balance across the stratification factors dynamically as clinicians are enrolled[29].

Randomization will be assigned on a 1:1 basis across arms. Though we will not be able to blind parents and clinicians to use of the decision aid, we will blind the investigators and all nonclinical stakeholders to study arm.

4.8.5 Healthcare Utilization

ED utilization will be estimated based on hospital billing data. Itemized hospital bills and UB-92 or UB-04 forms (summary billing statements) will be obtained for all patients for the 7-day period after the index visit. The UB-92 discharge form provides data on the patient encounters including demographic characteristics, discharge diagnoses, and discharge status (including death, but not its cause). For the economic analysis we will compare healthcare utilization descriptively and using multivariable models. For descriptive comparison of 7-day utilization we will utilize the Wilcoxon rank-sum test to account for the skewness of the outcome measures. We will estimate resource utilization using multivariable models, using the two-part or one-part generalized linear regression models. We will use a Park test to assess the appropriate distribution[30]; use a two-part model for outcomes where more than 10 percent of the subjects have zero outcome measure. The covariates in the multivariable models will include factors that may not be accounted for in the randomization such as type of healthcare insurance and severity of illness. We will also analyze the results using a subset of patients who primarily receive their entire healthcare at the participating centers. We expect that this analysis will provide a sensitivity analysis of the overall trial results based on self-report.

5.0 Conflict of Interest

Tools under evaluation are not part of any existing effort to commercialize or profit from their use; the researchers involved in this study have not received -- and will not receive with their application in this study -- any royalties or other monetary benefits, directly or indirectly, from use of the decision aids.

6.0 Human Subjects

6.1 Study monitoring

Monitoring for protocol adherence will be performed monthly during investigative steering committee conference calls to ensure early identification of poor performance at individual sites and in the trial overall. Specific parameters to be monitored will include randomization of ineligible subjects and treatment allocation errors (patients receiving the wrong intervention). The study team (PI, Co-PI’s, Lead study coordinator and the statistician) will track these events.

6.1.1 Data Safety Monitoring Plan

An independent Data Safety Monitoring Board has been convened . See "Data Safety Monitoring Plan."

6.2 Early Termination

This study will not be monitored for early termination due to benefit or futility. The intervention is an educational tool for use during the ED visit to help parents, along with their treating clinician, determine whether they wish to have their child have a head CT or not. The tool does not make recommendations or result in tests/treatments without the participation of the clinician, and the tool is to be used during a clinical encounter in which the clinician can place the information in context.

6.3 Data management

All sites will be required to use the current version of all documents and forms and adhere to the study schedule. Study sites will transcribe subject source data into eCRFs using REDCap for registration and data collection. The REDCap system is a HIPAA compliant secure data entry system that allows for validated data entry, edit checks and logs of all data changes.

All subsequent data (parent eligibility criteria, EMR review, parent survey (pre and post encounter), clinician survey and the parent 7 day follow-up) will be captured in the REDCap system by the site study coordinator.. A copy of the signed consent, case report form assessing parent/child eligibility, where the clinician signs-off along with copies of all surveys (parent and clinician) will be uploaded into REDCap as a PDF or word document or sent into the coordinating center, if the site does not have the capability to scan and upload files, and transferred into electronic storage behind the Mayo Clinic firewall in the study folder where only approved staff has access. All paper copies provided to the coordinating center will be destroyed after the transfer has been completed.

The data within REDCap can be accessed by the statistical team at any time and downloaded into a statistical software package. The statistical team will review the data on a monthly basis to ensure data accuracy and completeness. All data, documents, and analysis findings will be housed within the Mayo Clinic system that is password protected and backed up on a nightly basis. The data will be stored within the secure system for seven years following completion of the study.

6.4 Video and audio-recordings

Encounters will be video recorded where permission of all participants is obtained. These recordings are conducted using a portable hand-held digital video camera. Digital recordings will be immediately uploaded to the research team's secure server and deleted from portable devices after overnight back-up. The video and audio files are identified using a code number that does not include the name of the clinician, support staff, or patient or reference to their medical record number or date of birth. All transcriptions omit names that may have been stated during the recording, which are replaced in the transcript by purposefully false initials. The research data will only be accessible with password protected and logged access at the Mayo Clinic; all personnel have received human subjects research training. Audio and video files from facilities outside of Mayo Clinic, will be downloaded onto a password protected flash drive and forwarded to the Mayo research team via Fed Ex. On receiving the flash drives, the Mayo research team will immediately download the thumb drive to the Mayo server, where data are only accessible with password protected and logged access at Mayo Clinic. The video and audio files will be maintained at Mayo Clinic for purposes of future research and/or educational projects, if clinician and parent have specifically provided consent to do so.

6.5 Inclusion of Women and Minorities

**TARGETED/PLANNED ENROLLMENT: Number of Subjects**			
**Ethnic categories**	**Sex/Gender**		
	**Females**	**Males**	**Total**
Hispanic or Latino	67	66	133
Not Hispanic or Latino	408	409	817
**Ethnic Category: Total of All Subjects**	475	475	950
**Racial Categories**			
American Indian/Alaska Native (1%)	5	5	10
Asian (3%)	14	14	28
Black or African American (34%)	162	162	324
Native Hawaiian or other Pacific Islander (2%)	9	9	18
White (60%)	285	285	570
**Racial Categories: Total of All Subjects**	475	475	950

References

1. Langlois JA, Rutland-Brown W, Thomas KE. Traumatic brain injury in the united states: Emergency department visits, hospitalizations, and deaths 2002-2006. *In: CDC, ed. National Center for Injury Prevention and Control*. 2006
2. National center for injury prevention and control. Traumatic brain injury in the united states: Assessing outcomes in children. 2006;2012
3. Kuppermann N Fau - Holmes JF, Holmes Jf Fau - Dayan PS, Dayan Ps Fau - Hoyle JD, Jr., Hoyle Jd Jr Fau - Atabaki SM, Atabaki Sm Fau - Holubkov R, Holubkov R Fau - Nadel FM, Nadel Fm Fau - Monroe D, Monroe D Fau - Stanley RM, Stanley Rm Fau - Borgialli DA, Borgialli Da Fau - Badawy MK, Badawy Mk Fau - Schunk JE, Schunk Je Fau - Quayle KS, Quayle Ks Fau - Mahajan P, Mahajan P Fau - Lichenstein R, Lichenstein R Fau - Lillis KA, Lillis Ka Fau - Tunik MG, Tunik Mg Fau - Jacobs ES, Jacobs Es Fau - Callahan JM, Callahan Jm Fau - Gorelick MH, Gorelick Mh Fau - Glass TF, Glass Tf Fau - Lee LK, Lee Lk Fau - Bachman MC, Bachman Mc Fau - Cooper A, Cooper A Fau - Powell EC, Powell Ec Fau - Gerardi MJ, Gerardi Mj Fau - Melville KA, Melville Ka Fau - Muizelaar JP, Muizelaar Jp Fau - Wisner DH, Wisner Dh Fau - Zuspan SJ, Zuspan Sj Fau - Dean JM, Dean Jm Fau - Wootton-Gorges SL, Wootton-Gorges SL. Identification of children at very low risk of clinically-important brain injuries after head trauma: A prospective cohort study. *Lancet*. 2009;374:1160-1170
4. Pearce MS, Salotti JA, Little MP, McHugh K, Lee C, Kim KP, Howe NL, Ronckers CM, Rajaraman P, Craft AW, Parker L, Berrington de Gonz·lez A. Radiation exposure from ct scans in childhood and subsequent risk of leukaemia and brain tumours: A retrospective cohort study. *The Lancet*. 2012;380:499-505
5. Brenner D, Elliston C, Hall E, Berdon W. Estimated risks of radiation-induced fatal cancer from pediatric ct. *AJR Am J Roentgenol*. 2001;176:289-296
6. Smith-Bindman R, Lipson J, Marcus R, Kim KP, Mahesh M, Gould R, Berrington de Gonzalez A, Miglioretti DL. Radiation dose associated with common computed tomography examinations and the associated lifetime attributable risk of cancer. *Arch Intern Med*. 2009;169:2078-2086
7. Preston DL, Ron E, Tokuoka S, Funamoto S, Nishi N, Soda M, Mabuchi K, Kodama K. Solid cancer incidence in atomic bomb survivors: 1958-1998. *Radiat Res*. 2007;168:1-64
8. National cancer institute at the national institutes of health. Radiation risks and pediatric computed tomography (ct): A guide for health care providers. 2012;2012
9. Dunning J, Daly JP, Lomas JP, Lecky F, Batchelor J, Mackway-Jones K. Derivation of the children's head injury algorithm for the prediction of important clinical events decision rule for head injury in children. *Arch Dis Child*. 2006;91:885-891
10. Palchak MJ, Holmes JF, Vance CW, Gelber RE, Schauer BA, Harrison MJ, Willis-Shore J, Wootton-Gorges SL, Derlet RW, Kuppermann N. A decision rule for identifying children at low risk for brain injuries after blunt head trauma. *Ann Emerg Med*. 2003;42:492-506

11. Haydel MJ, Shembekar AD. Prediction of intracranial injury in children aged five years and older with loss of consciousness after minor head injury due to nontrivial mechanisms. *Ann Emerg Med*. 2003;42:507-514
12. Oman JA, Cooper RJ, Holmes JF, Viccellio P, Nyce A, Ross SE, Hoffman JR, Mower WR. Performance of a decision rule to predict need for computed tomography among children with blunt head trauma. *Pediatrics*. 2006;117:e238-246
13. Atabaki SM, Stiell IG, Bazarian JJ, Sadow KE, Vu TT, Camarca MA, Berns S, Chamberlain JM. A clinical decision rule for cranial computed tomography in minor pediatric head trauma. *Arch Pediatr Adolesc Med*. 2008;162:439-445
14. Osmond Mh Fau - Klassen TP, Klassen Tp Fau - Wells GA, Wells Ga Fau - Correll R, Correll R Fau - Jarvis A, Jarvis A Fau - Joubert G, Joubert G Fau - Bailey B, Bailey B Fau - Chauvin-Kimoff L, Chauvin-Kimoff L Fau - Pusic M, Pusic M Fau - McConnell D, McConnell D Fau - Nijssen-Jordan C, Nijssen-Jordan C Fau - Silver N, Silver N Fau - Taylor B, Taylor B Fau - Stiell IG, Stiell IG. Catch: A clinical decision rule for the use of computed tomography in children with minor head injury.
15. McGinn TG, Guyatt GH, Wyer PC, Naylor CD, Stiell IG, Richardson WS. Users' guides to the medical literature: Xxii: How to use articles about clinical decision rules. Evidence-based medicine working group. *JAMA*. 2000;284:79-84
16. Flynn D, Knoedler MA, Hess EP, Murad MH, Erwin PJ, Montori VM, Thomson RG. Engaging patients in health care decisions in the emergency department through shared decision-making: A systematic review. *Acad Emerg Med*. 2012;19:959-967
17. Yamamoto LG. Application of informed consent principles in the emergency department evaluation of febrile children at risk for occult bacteremia. *Hawaii Med J*. 1997;56:313-317, 320-312
18. Yamamoto LG, Young LL, Roberts JL. Informed consent and parental choice of anesthesia and sedation for the repair of small lacerations in children. *Am J Emerg Med*. 1997;15:285-289
19. Karpas A, Finkelstein M, Reid S. Parental preference for rehydration method for children in the emergency department. *Pediatr Emerg Care*. 2009;25:301-306
20. Hess EP, Knoedler MA, Shah ND, Kline JA, Breslin M, Branda ME, Pencille LJ, Asplin BR, Nestler DM, Sadosty AT, Stiell IG, Ting HH, Montori VM. The chest pain choice decision aid: A randomized trial. *Circ Cardiovasc Qual Outcomes*. 2012;5:251-259
21. Degner LF, Sloan JA, Venkatesh P. The control preferences scale. *Can J Nurs Res*. 1997;29:21-43
22. Elwyn G, Hutchings H, Edwards A, Rapport F, Wensing M, Cheung WY, Grol R. The option scale: Measuring the extent that clinicians involve patients in decision-making tasks. *Health Expect*. 2005;8:34-42
23. O'Connor AM. Validation of a decisional conflict scale. *Med Decis Making*. 1995;15:25-30

24. Koedoot N, Molenaar S, Oosterveld P, Bakker P, de Graeff A, Nooy M, Varekamp I, de Haes H. The decisional conflict scale: Further validation in two samples of dutch oncology patients. *Patient Educ Couns*. 2001;45:187-193
25. Anderson LA, Dedrick RF. Development of the trust in physician scale: A measure to assess interpersonal trust in patient-physician relationships. *Psychol Rep*. 1990;67:1091-1100
26. Thom DH, Ribisl KM, Stewart AL, Luke DA. Further validation and reliability testing of the trust in physician scale. The stanford trust study physicians. *Med Care*. 1999;37:510-517
27. IBID
28. Snijders TAB, Bosker RJ. Multilevel Analysis. An introduction to basic and advanced multilevel modelling. 2nd edition. 2012 Sage. Los Angeles
29. Pocock SJ, Simon R. Sequential treatment assignment with balancing for prognostic factors in controlled clinical trial. Biometrics 1975; 31, 103-115
30. Manning WG, Mullahy J. Estimating log models: to transform or not to transform? J Health Econ 2001;20:461-94.
